# Association between organophosphate pesticide exposure and atopic dermatitis: a cross-sectional study based on NHANES 1999–2007

**DOI:** 10.3389/fpubh.2025.1555731

**Published:** 2025-03-06

**Authors:** YueHua Men, YiMeng Wang, WenTing Wu, Ming Chu

**Affiliations:** ^1^Department of Dermatology, Peking University Third Hospital, Beijing, China; ^2^Department of Immunology, School of Basic Medical Sciences, NHC Key Laboratory of Medical Immunology, Peking University, Beijing, China

**Keywords:** organophosphate pesticides, atopic dermatitis, NHANES, environmental exposure, dialkyl phosphate metabolites, epidemiology

## Abstract

**Background:**

Organophosphate pesticides (OPPs) are widely used environmental chemicals with potential health impacts, but their relationship with atopic dermatitis (AD) remains unclear.

**Methods:**

Using data from the National Health and Nutrition Examination Survey (NHANES) 1999–2007, we investigated associations between urinary OPP metabolites and AD in 4,258 adults. Six dialkyl phosphate (DAP) metabolites were measured, and weighted quantile sum (WQS) regression was used to assess mixture effects.

**Results:**

Both DMP (odds ratio [OR] = 1.17, 95% confidence interval [CI]: 1.05–1.31) and DMDTP (OR = 2.23, 95%CI: 1.08–4.60) showed significant positive associations with AD in fully adjusted models. WQS regression revealed significant associations between mixed OPP exposure and AD (OR = 1.25, 95%CI: 1.04–1.50), with DMP contributing most (45.8%) to the mixture effect. Stratified analyses indicated stronger associations in males, younger adults (<60 years), and smokers.

**Conclusion:**

Our findings suggest that OPP exposure, particularly DMP, may be associated with increased AD risk in adults. These results provide new insights into environmental risk factors for AD.

## Introduction

Atopic dermatitis (AD) is a chronic inflammatory skin condition characterized by intense itching, skin barrier dysfunction and immune dysregulation (1). It represents a significant global health challenge, affecting approximately 2 billion people worldwide with a prevalence of 2.62% (2). The condition demonstrates notable demographic variations, with higher prevalence in children (3.96%) compared to adults (1.95%), and females (2.80%) versus males (2.44%) (3). Epidemiological studies show that the one-year prevalence ranges from 1.2 to 17.1% in adults and 0.96 to 22.6% in children (3). Particularly in developing regions, pediatric AD cases increased significantly between 1990 and 2019, with economically disadvantaged regions experiencing a 96.77% increase (4). Beyond its physical manifestations, AD significantly impacts patients’ quality of life, causing sleep disturbance, psychological distress, and social isolation, which substantially affects both patients and their families (5). This burden is further complicated by healthcare access disparities, with significant barriers in treatment accessibility, including registration challenges, costs, and insurance coverage issues (6).

The etiology of AD involves multiple factors, characterized by complex interactions between genetic and environmental factors. In terms of genetics, studies have identified over 30 genetic loci associated with AD, primarily involving skin barrier function and immune regulation, with loss-of-function mutations in the filaggrin gene representing the strongest known genetic risk factor (7, 8). Additionally, research has shown that parental history of atopic disease significantly increases the risk of AD in offspring (9). Beyond genetic factors, environmental factors also play crucial roles in both pathogenesis and clinical outcomes, including hygiene conditions, microbiota, endotoxin exposure, animal exposure, pollution, weather conditions, and dietary factors (10). Among various environmental exposures, organophosphorus pesticides (OPPs) have emerged as a subject of particular concern due to their widespread use and potential health implications. These compounds, commonly used in agricultural practices, demonstrate remarkable persistence in both terrestrial and aqueous environments (11).

The potential threat of OPPs to human health warrants significant attention, primarily due to their ability to modulate immune system function through multiple mechanisms (12, 13). These compounds mainly regulate inflammatory responses through the cholinergic pathway by interfering with cholinesterase activity, thereby affecting cytokine production and the expression of pro-inflammatory molecules (14). Experimental studies have demonstrated that OPP exposure can induce oxidative stress responses, leading to adverse effects on various biological systems, including immune function (15). Monitoring of human biological samples including urine has revealed the widespread presence of these compounds, indicating that populations are commonly exposed to OPPs (16). More importantly, research has found that both acute and chronic exposure to OPPs may induce chronic degenerative pathological changes and persistent inflammatory conditions (14), and this immune system dysregulation further highlights the potential health risks of OPP exposure.

Epidemiological studies have confirmed associations between pesticide exposure and various immune-mediated diseases. Research shows that pesticide exposure is significantly associated with increased risks of autoimmune diseases such as rheumatoid arthritis (17) and systemic lupus erythematosus (18). As an important class of pesticides, the relationship between OPPs and AD remains largely unexplored. This knowledge gap is particularly concerning given several critical factors. First, the increasing global use of OPPs, especially in developing countries, raises concerns about their potential contribution to the rising prevalence of AD (19). Second, while acute toxicity of OPPs is well documented, their chronic effects on immune-mediated diseases remain poorly understood (20). Third, identifying environmental risk factors for AD is crucial for developing prevention strategies (21), particularly in populations with high pesticide exposure. The potential impact of OPPs on allergic diseases represents a significant public health concern that requires urgent investigation (22).

Therefore, this study is dedicated to elucidating this potential connection. Our primary research questions were: (1) Is there an association between OPP exposure and AD risk in the general adult population? (2) Do different OPP metabolites contribute differently to AD risk? (3) Are there specific population subgroups more susceptible to OPP-associated AD risk? Based on previous evidence showing OPPs’ effects on immune function and inflammatory responses, we hypothesized that increased OPP exposure would be associated with higher AD risk, and this association might vary across different population subgroups due to variations in metabolic capacity. This study aims to provide new clinical evidence for optimizing prevention strategies and treatment approaches for AD.

## Methods

### Study population and data source

The National Health and Nutrition Examination Survey (NHANES) is a nationally representative survey conducted by the National Center for Health Statistics (NCHS) under the Centers for Disease Control and Prevention (CDC), designed to assess the health and nutritional status of the U.S. population. NHANES employs a complex multistage stratified probability sampling method, with biennial cycles, selecting representative samples from counties across the United States and conducting comprehensive health and nutrition assessments. Based on the concurrent availability of data on OPP metabolites and AD, this study utilized data from four survey cycles: 1999–2000, 2001–2002, 2003–2004, and 2005–2007. These data are publicly accessible through the CDC website.

**Figure 1:**
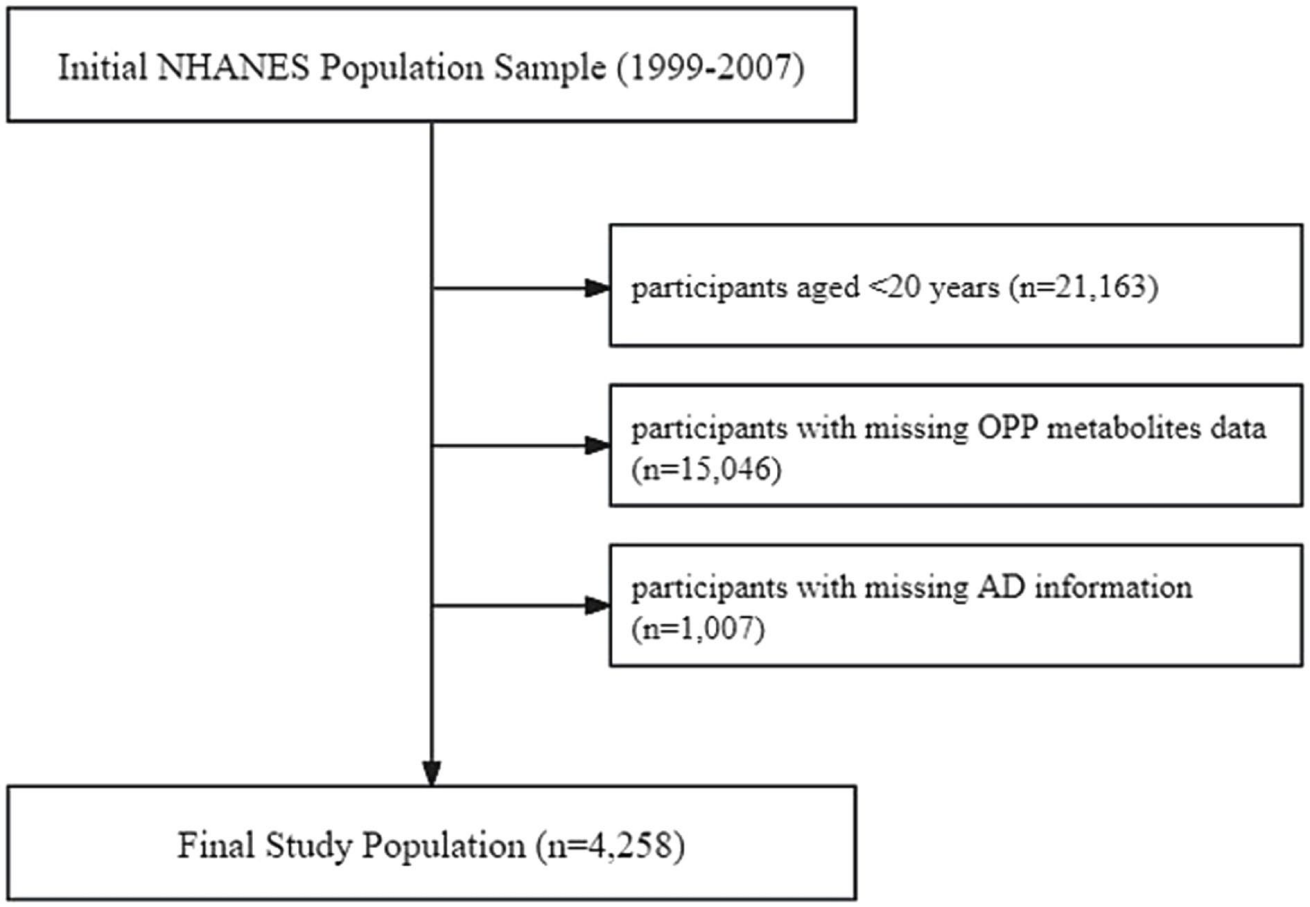
Flow chart of participant selection from the NHANES, 1999–2007.

The exclusion criteria for study subjects, as shown in Figure 1, included: (1) age < 20 years (*n* = 21,163); (2) missing data on OPP metabolites (*n* = 15,046); and (3) missing information on AD (*n* = 1,007). A total of 4,258 participants were ultimately included in the analysis. All participants provided written informed consent, and the study protocol was approved by the NCHS Research Ethics Review Board.

### Definition of AD

Atopic dermatitis was identified based on self-reported data from the NHANES questionnaires. Three questions were used from the Allergy and Dermatology Questionnaires: “Has a doctor or other health professional ever told you that you have eczema?,” “Do you have dermatitis or eczema today?,” and “During the past 12 months, have you had dermatitis, eczema, or any other type of red, inflamed skin rash?.” Participants who answered “yes” to any of these questions were defined as having AD. All questions were derived from standardized NHANES questionnaire modules that underwent rigorous pretesting and validation.

### Measurement of OPPs

Urinary concentrations of six dialkyl phosphate (DAP) metabolites were measured as biomarkers of OPP exposure. These metabolites included dimethylphosphate (DMP), diethylphosphate (DEP), dimethylthiophosphate (DMTP), diethylthiophosphate (DETP), dimethyldithiophosphate (DMDTP), and diethyldithiophosphate (DEDTP). Urine samples were collected from participants as single spot samples and analyzed at the National Center for Environmental Health (NCEH), CDC (Atlanta, Georgia). While spot urine samples are commonly used in large epidemiological studies for practical and feasibility reasons (23). The analysis was performed using solid phase extraction coupled with ultrahigh-performance liquid chromatography–tandem mass spectrometry (UPLC-MS/MS). For values below the lower limit of detection (LOD), LOD divided by the square root of two was used as a replacement, following standard practice in environmental exposure assessment (24). All analyses were performed following standardized NHANES laboratory procedures and quality control protocols.

### Covariates

Potential confounding factors included demographic characteristics (age, sex, race, education level, and poverty income ratio), lifestyle factors (smoking status, alcohol consumption, and physical activity), chronic diseases (hypertension and diabetes), and dietary inflammatory index (DII). Race was categorized into Mexican American, Other Hispanic, Non-Hispanic White, Non-Hispanic Black, and Other Race. Education level was classified into five categories from lowest to highest: less than 9th grade, 9-11th grade, high school graduate, some college, and college graduate or above. Smokers were defined as those who had smoked at least 100 cigarettes in their lifetime, and alcohol users were defined as those who had at least 12 drinks in the past year. Participants were considered to have hypertension if they had a doctor’s diagnosis, used antihypertensive medication, or had blood pressure ≥ 140/90 mmHg. Diabetes was defined by a doctor’s diagnosis, HbA1c ≥6.5%, fasting glucose ≥7.0 mmol/L, random glucose ≥11.1 mmol/L, 2-h OGTT glucose ≥11.1 mmol/L, or use of antidiabetic medication or insulin. Physical activity level was calculated based on the frequency and duration of moderate and vigorous activities related to work, recreation, and transportation from NHANES questionnaires, following previously validated methodology (25). All analyses were adjusted for log-transformed urinary creatinine.

### Statistical analysis

To account for NHANES’ representativeness, appropriate sample weights, strata, and cluster variables were employed according to its complex multistage sampling design. Eight-year weights were calculated according to NHANES guidelines as the data spanned four survey cycles (1999–2007). Due to substantial right-skewed distributions, urinary creatinine-corrected concentrations of six OPP metabolites were natural log (ln) transformed and categorized into quartiles (Q1–Q4). Continuous variables were presented as weighted means ± standard errors, and categorical variables as counts with weighted percentages. Weighted *t*-tests and chi-square tests were used to compare baseline characteristics between AD and non-AD groups.

For correlation analysis, Pearson correlation coefficients were used to assess the strength of associations among six DAPs (|*r*| < 0.3, 0.3–0.7, and ≥0.7 indicating weak, moderate, and strong correlations, respectively). The main analysis employed survey-weighted generalized linear regression models (SWGLMs) to evaluate associations between OPP metabolites (as both continuous variables and quartiles) and AD, with *P* for trend calculated for quartile analyses. Three progressively adjusted models were constructed: Model 1 adjusted for ln-transformed urinary creatinine; Model 2 further adjusted for demographic characteristics (age, sex, race, poverty, and education); Model 3 additionally adjusted for lifestyle and health conditions (smoking, alcohol consumption, hypertension, diabetes, DII, and physical activity). Furthermore, to explore potential non-linear associations between OPP metabolites and AD, smoothing curve fitting analysis was performed using generalized additive models (GAM) with adjustment for all covariates in Model 3.

To assess the combined effects of OPP exposure on AD, we employed the Weighted Quantile Sum (WQS) regression model, a statistical method specifically designed for evaluating health outcomes associated with multiple chemical exposures (26). This approach generates a weighted index to assess overall effects while determining each compound’s relative contribution. The original dataset was randomly split into training and validation sets (40: 60 ratio), with 1,000 bootstrap iterations to ensure result stability. Weights were optimized in the training set and validated in the test set. The WQS model adjusted for all covariates from Model 3, including ln-transformed urinary creatinine, demographic characteristics, lifestyle factors, and chronic conditions. Each OPP metabolite was assigned a weight between 0 and 1, with the sum of weights equaling 1, where higher weights indicated greater contributions to AD risk. This analysis enabled simultaneous evaluation of the six OPP metabolites’ mixed exposure effects and identification of key metabolites contributing most significantly to AD risk.

Finally, subgroup analyses and interaction tests were performed for OPP metabolites significantly associated with AD. Analyses were stratified by sex, age (≥60 or <60 years), smoking status, alcohol consumption, hypertension, and diabetes, adjusting for all covariates in Model 3.

All statistical analyses were performed using R version 4.3.2, with the “survey” package for weighted analysis and “gWQS” for WQS regression. Two-sided *p* < 0.05 was considered statistically significant.

## Results

### Baseline characteristics of study population

**Figure 2:**
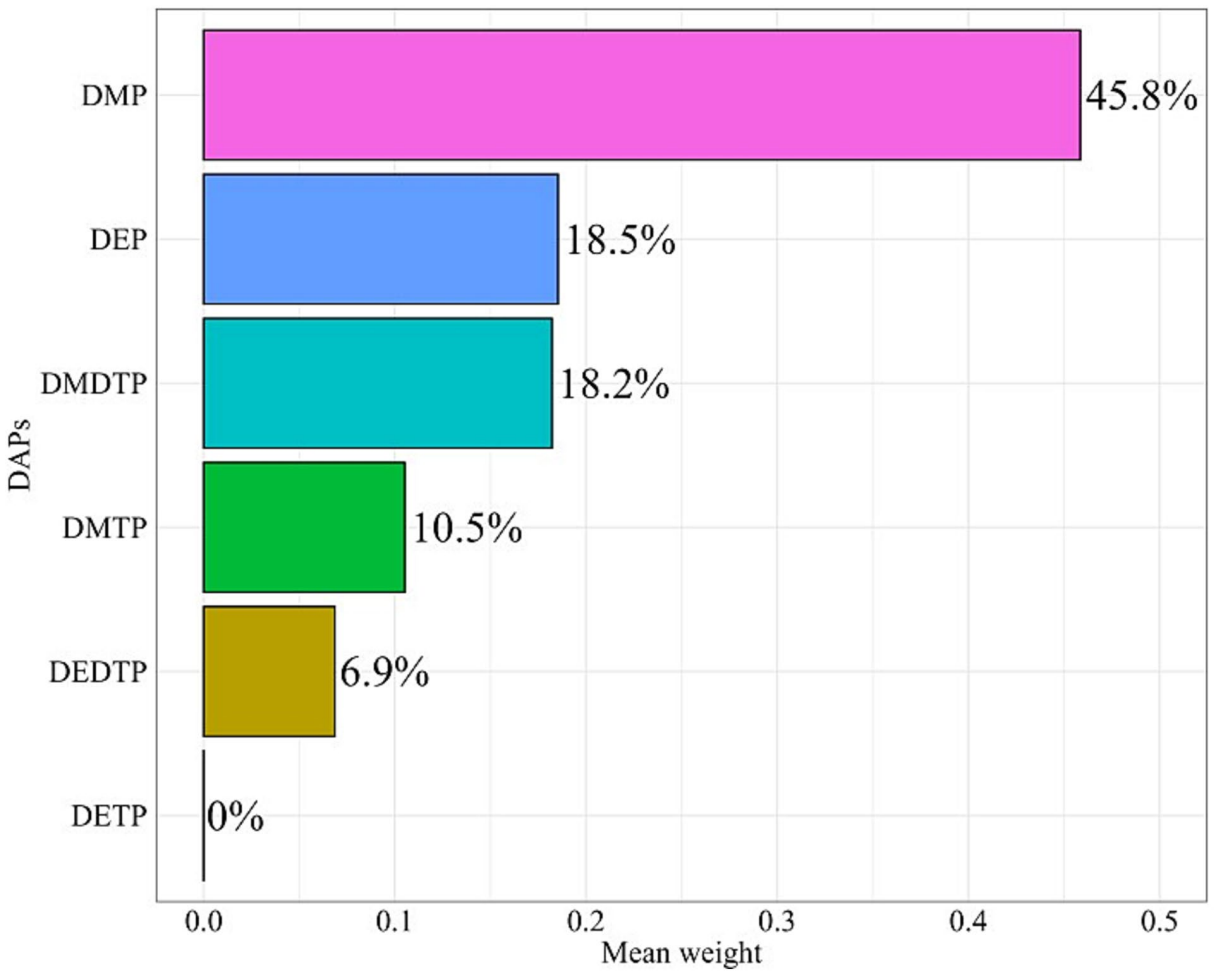
Correlation matrix of DAP metabolites among study participants. DMP, dimethylphosphate; DEP, diethylphosphate; DMTP, dimethylthiophosphate; DETP, diethylthiophosphate; DMDTP, dimethyldithiophosphate; DEDTP, diethyldithiophosphate.

A total of 4,258 participants were included in the final analysis, with 280 (6.6%) diagnosed with AD. Compared to the non-AD group, AD patients showed a significantly higher proportion of females (60.17% vs. 49.16%, *p* = 0.008) and were predominantly Non-Hispanic White (76.07%). No significant differences were observed in age (41.93 ± 0.90 vs. 41.33 ± 0.38 years) or poverty income ratio between groups (Table 1). AD patients reported significantly lower physical activity levels (206.81 ± 18.14 vs. 253.13 ± 10.15 h/week, *p* = 0.034), while dietary inflammatory index showed no significant difference between groups (1.30 ± 0.14 vs. 1.36 ± 0.04, *p* = 0.719). Regarding OPP metabolite levels, the AD group exhibited significantly higher levels of DMP (0.54 ± 0.10 vs. 0.22 ± 0.04, *p* = 0.002), DEP (0.07 ± 0.11 vs. −0.17 ± 0.06, *p* = 0.024), DMTP (0.82 ± 0.14 vs. 0.51 ± 0.05, *p* = 0.032), and DMDTP (−0.66 ± 0.12 vs. −0.91 ± 0.05, *p* = 0.031). Correlation analysis revealed moderate correlations (0.3 ≤ |*r*| < 0.7) among all six DAP metabolites, with the strongest correlations observed between DMTP and DMDTP (*r* = 0.51), followed by DMP and DMTP (*r* = 0.44), and DETP and DEDTP (*r* = 0.43) (Figure 2).

**Table 1:** Baseline characteristics of study population according to AD status.

Variables	AD		*p*-value
	No	Yes	
Age (years)	41.33 ± 0.38	41.93 ± 0.90	0.559
Poverty income ratio	3.08 ± 0.05	3.09 ± 0.14	0.950
ln (DMP, μg/L)	0.22 ± 0.04	0.54 ± 0.10	0.002
ln (DEP, μg/L)	−0.17 ± 0.06	0.07 ± 0.11	0.024
ln (DMTP, μg/L)	0.51 ± 0.05	0.82 ± 0.14	0.032
ln (DETP, μg/L)	−0.71 ± 0.06	−0.57 ± 0.09	0.083
ln (DMDTP, μg/L)	−0.91 ± 0.05	−0.66 ± 0.12	0.031
ln (DEDTP, μg/L)	−1.54 ± 0.04	−1.44 ± 0.08	0.197
ln (urinary creatinine, mg/dL)	4.62 ± 0.02	4.68 ± 0.04	0.166
DII	1.36 ± 0.04	1.30 ± 0.14	0.719
PA (hours/week)	253.13 ± 10.15	206.81 ± 18.14	0.034
Sex			0.008
Male	1896 (50.84%)	117 (39.83%)	
Female	2082 (49.16%)	163 (60.17%)	
Race			0.007
Mexican American	930 (8.63%)	30 (3.80%)	
Other Hispanic	186 (5.40%)	12 (3.37%)	
Non-Hispanic White	1847 (69.00%)	164 (76.07%)	
Non-Hispanic Black	838 (11.27%)	58 (10.06%)	
Other Race	177 (5.74%)	16 (6.70%)	
Education level			0.110
Less than 9th grade	447 (5.71%)	12 (2.59%)	
9–11th grade	676 (12.44%)	33 (8.57%)	
High school graduate	938 (24.61%)	62 (24.75%)	
College	1,127 (31.34%)	104 (35.93%)	
College graduate or above	790 (25.91%)	69 (28.17%)	
Smoke			0.140
No	2,142 (51.59%)	128 (45.76%)	
Yes	1836 (48.41%)	152 (54.24%)	
Alcohol consumption			0.175
No	1,101 (25.74%)	62 (21.78%)	
Yes	2,595 (74.26%)	199 (78.22%)	
Hypertension			0.951
No	3,003 (77.02%)	210 (77.22%)	
Yes	943 (22.98%)	70 (22.78%)	
Diabetes			0.218
No	3,698 (94.52%)	268 (96.38%)	
Yes	280 (5.48%)	12 (3.62%)	

AD, atopic dermatitis; DMP, dimethylphosphate; DEP, diethylphosphate; DII, dietary inflammatory index; DMTP, dimethylthiophosphate; DETP, diethylthiophosphate; DMDTP, dimethyldithiophosphate; DEDTP, diethyldithiophosphate; PA, Physical activity.

### Association between individual DAP metabolites and AD

The SWGLMs were used to analyze associations between individual metabolites and AD. For DMP, significant associations were observed in Model 1 for both continuous exposure (OR = 1.18, 95%CI: 1.07–1.30, *p* = 0.002; *P* for trend = 0.011) and highest quartile (Q4 vs. Q1: OR = 1.61, 95%CI: 1.13–2.28, *p* = 0.008). These associations remained consistent in Model 2 (continuous: OR = 1.16, 95%CI: 1.04–1.30, *p* = 0.008; Q4 vs. Q1: OR = 1.54, 95%CI: 1.05–2.27, *p* = 0.029; *P* for trend = 0.028) and Model 3 (continuous: OR = 1.17, 95%CI: 1.05–1.31, *p* = 0.006; Q4 vs. Q1: OR = 1.61, 95%CI: 1.07–2.41, *p* = 0.024; *P* for trend = 0.030). DMDTP showed significant associations in Model 1 (continuous: OR = 1.10, 95%CI: 1.01–1.20, *p* = 0.029; Q4 vs. Q1: OR = 1.85, 95%CI: 1.02–3.35, *p* = 0.042; *P* for trend = 0.021), slightly attenuated in Model 2 (*P* for trend = 0.028), but regained significance in Model 3’s highest quartile (OR = 2.23, 95%CI: 1.08–4.60, *p* = 0.031; *P* for trend = 0.016). No significant associations were found between other metabolites (DEP, DMTP, DETP, and DEDTP) and AD risk (Table 2).

**Table 2:** Associations between DAP metabolites levels and AD in multivariable-adjusted models.

Variables	AD					
	Model 1		Model 2		Model 3	
	OR, 95% CI	*p*-value	OR, 95% CI	*P*-value	OR, 95% CI	*P*-value
DMP						
Continuous	1.18 (1.07, 1.30)	**0.002**	1.16 (1.04, 1.30)	**0.008**	1.17 (1.05, 1.31)	**0.006**
Q1	Reference	–	Reference	–	Reference	–
Q2	0.87 (0.49, 1.53)	0.620	0.87 (0.50, 1.53)	0.619	0.87 (0.47, 1.61)	0.655
Q3	1.05 (0.74, 1.49)	0.787	1.05 (0.73, 1.52)	0.787	1.03 (0.67, 1.58)	0.897
Q4	1.61 (1.13, 2.28)	**0.008**	1.54 (1.05, 2.27)	**0.029**	1.61 (1.07, 2.41)	**0.024**
*P* for trend	1.19 (1.04, 1.35)	**0.011**	1.17 (1.02, 1.34)	**0.028**	1.18 (1.02, 1.38)	**0.030**
DEP						
Continuous	1.10 (1.01, 1.20)	**0.032**	1.09 (0.99, 1.20)	**0.070**	1.08 (0.98, 1.18)	0.112
Q1	Reference	–	Reference	–	Reference	–
Q2	1.24 (0.74–2.08)	0.417	1.20 (0.70–2.07)	0.503	1.22 (0.69–2.17)	0.484
Q3	1.38 (0.87–2.18)	0.170	1.36 (0.84–2.21)	0.202	1.42 (0.88–2.27)	0.144
Q4	1.51 (0.94–2.43)	0.087	1.44 (0.87–2.38)	0.153	1.44 (0.83–2.50)	0.186
*P* for trend	1.14 (0.99–1.32)	0.076	1.13 (0.96–1.32)	0.132	1.13 (0.96–1.33)	0.139
DMTP						
Continuous	1.11 (1.00–1.23)	0.043	1.10 (0.99–1.22)	0.074	1.08 (0.98–1.20)	0.122
Q1	Reference	–	Reference	–	Reference	–
Q2	1.45 (0.91–2.29)	0.112	1.44 (0.90–2.29)	0.121	1.32 (0.80–2.16)	0.268
Q3	1.13 (0.70–1.82)	0.605	1.10 (0.66–1.82)	0.721	1.08 (0.63–1.87)	0.771
Q4	1.79 (1.13–2.82)	0.014	1.75 (1.09–2.81)	0.021	1.60 (0.99–2.58)	0.055
*P* for trend	1.16 (1.01–1.33)	0.033	1.15 (1.00–1.33)	0.054	1.13 (0.98–1.31)	0.099
DETP						
Continuous	1.10 (0.98–1.23)	0.114	1.11 (0.98–1.25)	0.089	1.11 (0.97–1.27)	0.142
Q1	Reference	–	Reference	–	Reference	–
Q2	1.18 (0.77–1.81)	0.437	1.16 (0.75–1.79)	0.496	1.20 (0.74–1.94)	0.461
Q3	1.11 (0.73–1.67)	0.629	1.10 (0.72–1.68)	0.636	1.03 (0.63–1.68)	0.893
Q4	1.42 (0.92–2.18)	0.11	1.46 (0.94–2.26)	0.093	1.52 (0.93–2.48)	0.092
*P* for trend	1.10 (0.96–1.27)	0.178	1.12 (0.96–1.29)	0.141	1.12 (0.95–1.32)	0.162
DMDTP						
Continuous	1.10 (1.01–1.20)	**0.029**	1.10 (1.00–1.20)	**0.047**	1.09 (0.99–1.21)	0.081
Q1	Reference	–	Reference	–	Reference	–
Q2	1.19 (0.71–2.02)	0.5	1.17 (0.68–2.04)	0.562	1.45 (0.76–2.75)	0.249
Q3	1.21 (0.63–2.31)	0.559	1.18 (0.60–2.33)	0.618	1.48 (0.68–3.20)	0.314
Q4	1.85 (1.02–3.35)	**0.042**	1.83 (0.97–3.46)	0.063	2.23 (1.08–4.60)	**0.031**
*P* for trend	1.22 (1.03–1.45)	**0.021**	1.22 (1.02–1.47)	**0.028**	1.26 (1.05–1.51)	**0.016**
DEDTP						
Continuous	1.11 (0.95–1.30)	0.197	1.11 (0.94–1.31)	0.222	1.15 (0.95–1.38)	0.139
Q1	Reference	–	Reference	–	Reference	–
Q2	0.95 (0.64–1.42)	0.811	1.00 (0.64–1.57)	0.991	1.15 (0.53–2.46)	0.719
Q3	1.16 (0.75–1.79)	0.504	1.19 (0.74–1.91)	0.466	1.28 (0.60–2.72)	0.516
Q4	1.20 (0.76–1.89)	0.422	1.23 (0.74–2.04)	0.422	1.48 (0.69–3.15)	0.306
*P* for trend	1.09 (0.94–1.26)	0.226	1.09 (0.93–1.27)	0.269	1.14 (0.95–1.35)	0.149

Bold values indicate statistically significant *p* values (*p* < 0.05).

Three models were constructed:

Model 1 adjusted for ln-transformed urinary creatinine.

Model 2 further adjusted for age, sex, race, poverty, and education.

Model 3 additionally adjusted for smoking, alcohol consumption, hypertension, diabetes, DII, and physical activity.

AD, atopic dermatitis; CI, confidence interval; DMP, dimethylphosphate; DEP, diethylphosphate; DMTP, dimethylthiophosphate; DETP, diethylthiophosphate; DMDTP, dimethyldithiophosphate; DEDTP, diethyldithiophosphate; OR, odds ratio.

**Figure 3:**
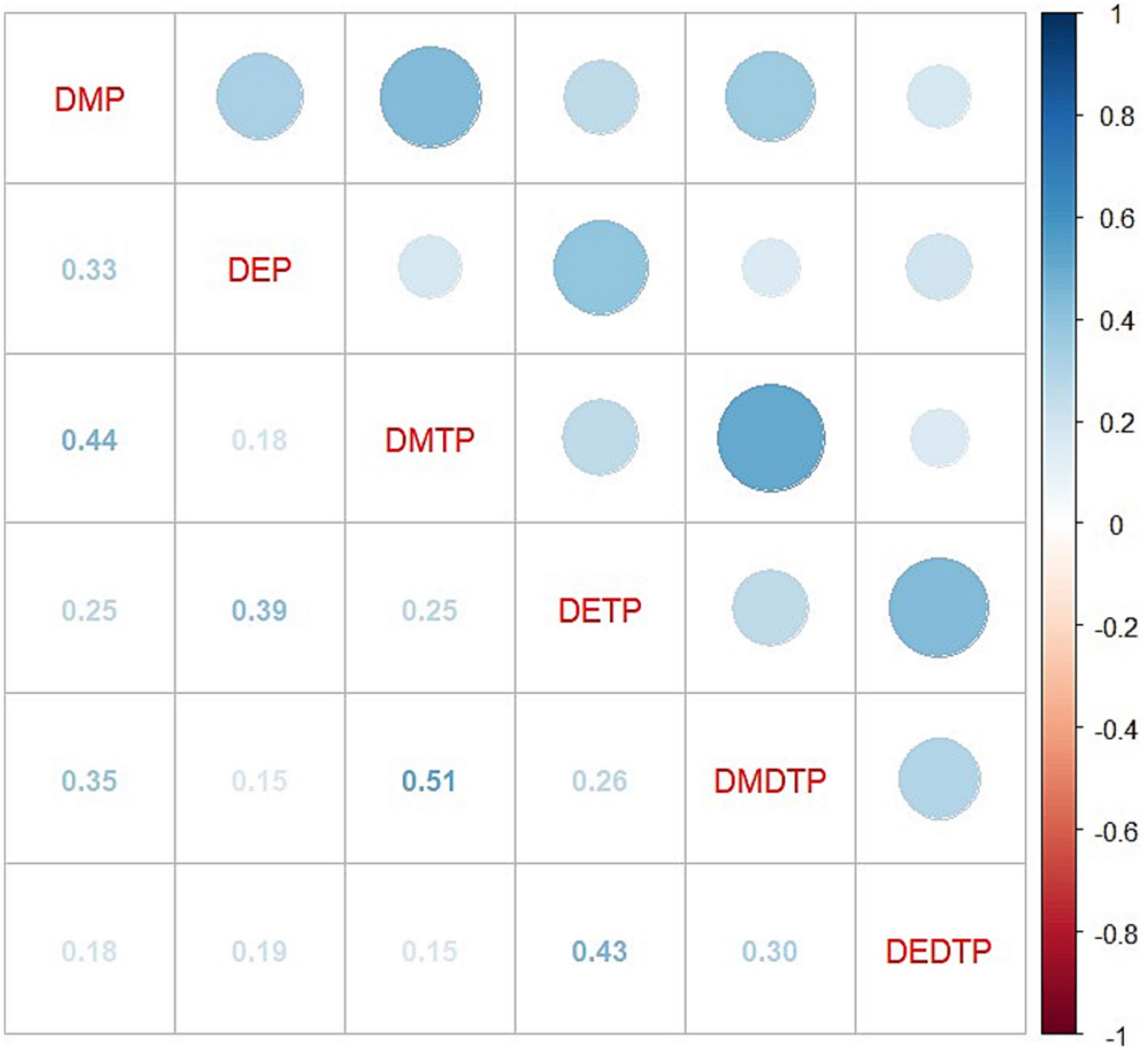
Smoothing plots of the associations between DAP metabolites and AD after adjustment for covariates. AD, atopic dermatitis; DMP, dimethylphosphate; DEP, diethylphosphate; DMTP, dimethylthiophosphate; DETP, diethylthiophosphate; DMDTP, dimethyldithiophosphate; DEDTP, diethyldithiophosphate.

To further explore the association patterns between DAP metabolites and AD, we performed smoothing curve fitting analysis (Figure 3). After adjusting for all potential confounding factors, DMP and DMDTP showed notable positive associations with AD risk, with AD risk increasing gradually as metabolite levels increased. The associations between other metabolites (DEP, DMTP, DETP, and DEDTP) and AD were relatively weak.

### Combined exposure effects of OPP metabolites

**Figure 4:**
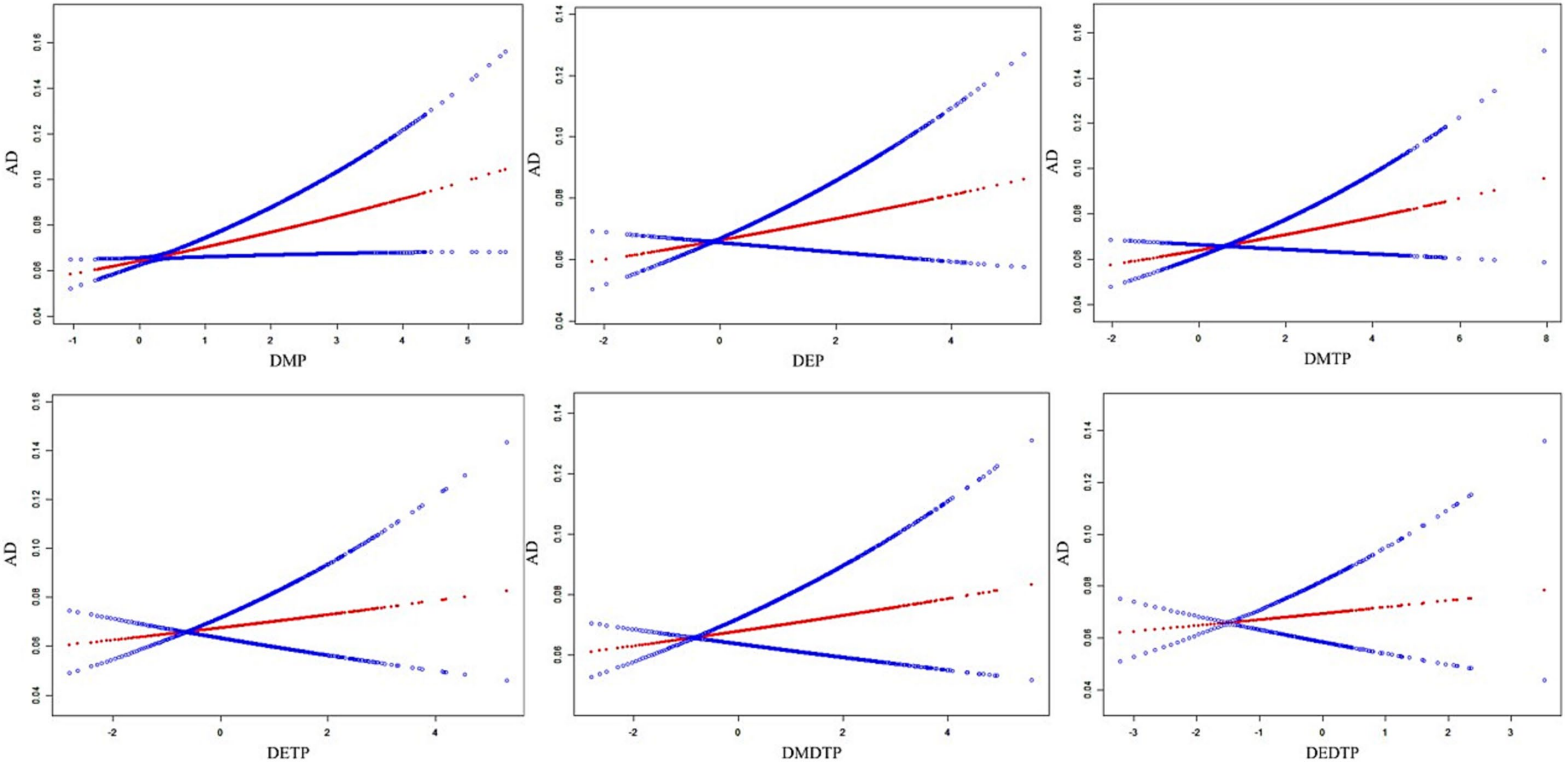
Weighted contribution of individual DAP metabolites in the WQS regression analysis. DMP, dimethylphosphate; DEP, diethylphosphate; DMTP, dimethylthiophosphate; DETP, diethylthiophosphate; DMDTP, dimethyldithiophosphate; DEDTP, diethyldithiophosphate.

Weighted quantile sum regression analysis adjusted for variables in Model 3 revealed a significant association between mixed exposure and AD risk (OR = 1.25, 95%CI: 1.04–1.50, *p* = 0.019). In the relative contribution analysis of six metabolites, DMP showed the highest weight (45.8%), followed by DEP (18.5%) and DMDTP (18.2%), while DMTP (10.5%) and DEDTP (6.9%) contributed relatively less, and DETP showed no contribution (0%) (Figure 4).

### Stratified and interaction analyses of DAP metabolites and AD

Dimethylphosphate and DMDTP were analyzed across subgroups of sex, age, smoking status, alcohol consumption, hypertension, and diabetes (Table 3). DMP showed significant positive associations with AD among males (OR = 1.22, 95%CI: 1.06–1.39, *p* = 0.004), age < 60 years (OR = 1.16, 95%CI: 1.05–1.27, *p* = 0.003), smokers (OR = 1.22, 95%CI: 1.08–1.38, *p* = 0.002), and alcohol consumers (OR = 1.15, 95%CI: 1.04–1.28, *p* = 0.008). No significant associations were found among females (OR = 1.01, 95%CI: 0.89–1.14, *p* = 0.899) and non-smokers (OR = 0.98, 95%CI: 0.85–1.12, *p* = 0.744), while a significant negative association was observed in age ≥ 60 years group (OR = 0.69, 95%CI: 0.48–1.00, *p* = 0.048). Significant interactions were found for sex (*P* for interaction = 0.045), age (*P* for interaction = 0.003), and smoking status (*P* for interaction = 0.017), indicating stronger associations in males versus females, positive association in younger (<60 years) versus negative in older (≥60 years) adults, and stronger association in smokers versus non-smokers. DMDTP showed no significant associations across subgroups and no significant interactions.

**Table 3:** Stratified and interaction analyses of associations between DMP, DMDTP, and AD according to participant characteristics.

Variables	OR 95%CI	*P*-value	*P* for interaction
DMP			
Sex			**0.045**
Male	1.22 (1.06, 1.39)	0.004	
Female	1.01 (0.89, 1.14)	0.899	
Age			**0.003**
<60 years	1.16 (1.05, 1.27)	0.003	
≥60 years	0.69 (0.48, 1.00)	0.048	
Smoke			**0.017**
No	0.98 (0.85, 1.12)	0.744	
Yes	1.22 (1.08, 1.38)	0.002	
Alcohol consumption			0.098
No	0.96 (0.80, 1.16)	0.683	
Yes	1.15 (1.04, 1.28)	0.008	
Hypertension			0.438
No	1.12 (1.01, 1.25)	0.030	
Yes	1.03 (0.86, 1.24)	0.724	
Diabetes			0.712
No	1.10 (1.00, 1.21)	0.041	
Yes	1.02 (0.67, 1.54)	0.933	
DMDTP			
Sex			0.595
Male	1.00 (0.88, 1.14)	0.954	
Female	1.05 (0.94, 1.17)	0.379	
Age			0.556
<60 years	1.05 (0.96, 1.14)	0.273	
≥60 years	0.86 (0.44, 1.70)	0.669	
Smoke			0.695
No	1.02 (0.90, 1.15)	0.749	
Yes	1.05 (0.94, 1.18)	0.373	
Alcohol consumption			0.428
No	0.98 (0.83, 1.16)	0.831	
Yes	1.06 (0.96, 1.17)	0.225	
Hypertension			0.588
No	1.05 (0.95, 1.15)	0.339	
Yes	0.99 (0.83, 1.19)	0.913	
Diabetes			0.945
No	1.03 (0.95, 1.13)	0.447	
Yes	1.02 (0.65, 1.59)	0.939	

Bold values indicate statistically significant *p* values (*p* < 0.05).AD, atopic dermatitis; CI, confidence interval; DMP, dimethylphosphate; DEP, diethylphosphate; DMTP, dimethylthiophosphate; DETP, diethylthiophosphate; DMDTP, dimethyldithiophosphate; DEDTP, diethyldithiophosphate; OR, odds ratio.

## Discussion

This study investigated the associations between six urinary OPP metabolites (DMP, DEP, DMTP, DETP, DMDTP, and DEDTP) and AD in the general adult population using cross-sectional data from NHANES 1999–2007. Our analysis revealed a positive correlation between DMP levels and the risk of AD. Based on multivariable-adjusted WQS regression analysis, exposure to the mixture of OPP metabolites was significantly associated with increased risk of AD, suggesting that OPP exposure might be a potential environmental risk factor for AD.

Previous studies have established various health effects of OPPs, primarily focusing on three key areas: endocrine disruption, metabolic effects, and neurotoxicity. Case reports have documented direct dermatological effects, with occupational exposure cases reporting contact dermatitis and skin irritation among agricultural workers handling OPPs (27). Studies examining chronic OPP exposure have also associated it with various skin manifestations (28). Research has shown that OPPs can disrupt endocrine function through multiple pathways, including effects on hormone biosynthesis, transport, receptor binding, decreased CRH expression with enhanced negative feedback, leading to HPA axis dysfunction and cortisol secretion abnormalities (29), and estrogen receptor activation resulting in adipogenesis (30–32). Furthermore, epidemiological studies have found that OPP exposure is significantly associated with metabolic disorders including hypertension, hyperglycemia, and dyslipidemia, and can cause gut microbiota dysbiosis, such as enrichment of pathogenic bacteria like Paraprevotella and Helicobacter while reducing beneficial flora, potentially contributing to the development of obesity and type 2 diabetes (33, 34). Additionally, research has demonstrated that OPP exposure, particularly during gestation, can induce both acute cholinesterase inhibition and chronic cognitive dysfunction, resulting in symptoms such as memory decline and anxiety-like behavior (35). More importantly, studies have found that OPPs can affect immune system function by inhibiting CD4+ and CD8+ T cell proliferation and reducing IL-2 secretion and CD25 expression (36). Despite these well-documented effects, there has been a notable gap in understanding the relationship between OPP exposure and allergic conditions, particularly AD, in the general population, which motivated our current study.

Atopic dermatitis is characterized by a complex pathophysiological mechanism involving three interconnected primary components: skin barrier dysfunction manifested as impaired stratum corneum integrity and increased transepidermal water loss, immune dysregulation encompassing both innate and adaptive immune response abnormalities, and pruritus closely associated with neuroimmune interactions (37). At the molecular level, AD pathogenesis involves multiple key immune pathways: Th2 cell-mediated IL-4 and IL-13 signaling triggering inflammation and IgE production (38), Langerhans cell-mediated responses through antigen presentation and cytokine secretion (39), autoimmune responses including IgE autoantibody production and autoreactive T cell activation (40), and Th2/Th17 cytokine imbalance mediated through JAK signaling (41). These immune alterations, together with genetic susceptibility, epidermal barrier defects, and skin microbiome dysbiosis, form the pathogenic basis of AD (42). The relationship between OPP metabolites and AD may be mediated through multiple mechanisms. Through rat experiments, Yang et al. found that DEP exposure affects thyroid hormone levels and increases inflammatory factors including TNF-α and IL-6, suggesting its influence on inflammatory responses through the endocrine-immune axis. Animal studies have shown that DEP exposure elevates Th2-type cytokines IL-4 and IL-13, changes associated with AD-like skin inflammation (43). Additionally, as reviewed by Freyre et al. (44), OPP metabolite-induced oxidative stress can activate inflammatory pathways, potentially promoting allergic diseases. Furthermore, OPPs may also directly affect skin health through dermal contact, as studies have shown that occupational or environmental dermal exposure to OPPs can cause direct skin irritation and inflammation (45), potentially contributing to various skin conditions including contact dermatitis (46). This direct irritant effect on the skin barrier might work synergistically with their systemic effects through immune and inflammatory pathways to increase AD risk (47).

The unique contribution of this study lies in using mixture analysis to evaluate the combined effects of six OPP metabolites, not only quantifying the individual impact strength of each metabolite but also revealing for the first time their synergistic effects on AD development. Our identification of DMP’s predominant role in the association between OPPs and AD provides a new perspective for understanding specific OPP metabolites in allergic conditions, extending beyond traditional research focused on acute toxicity and endocrine disruption. DMP is a common metabolite of several widely used organophosphate pesticides, including malathion, methyl parathion, and dichlorvos, which undergo biotransformation through demethylation processes in the human body (16). Notably, recent studies have successively found elevated DMP levels associated with various health issues: increased risk of depression (48), abnormal thyroid autoimmune indicators (49), and close correlation with asthma development (50). This evidence collectively suggests that DMP may play a central role in the pathogenesis of immune-mediated and endocrine diseases through its effects on inflammatory pathways and immune regulation. Given the widespread presence of OPPs in the environment and their significant biological effects, these findings have important public health implications for understanding prevention and intervention strategies for environment-related diseases.

Subgroup analyses revealed several important findings. Males showed higher DMP susceptibility than females, possibly due to sex differences in detoxifying enzyme activities like CYP450 and PON1 regulated by sex hormones (51). Detailed analysis of gender-specific lifestyle patterns revealed distinct differences in alcohol consumption between male and female AD patients (Supplementary Table 1). Female AD patients showed significantly lower rates of alcohol consumption (71.90% vs. 87.21% in males, *p* = 0.007), while no significant gender difference was observed in smoking status (52.37% vs. 57.06% in males, *p* = 0.441). These differences in alcohol consumption patterns might partially contribute to the gender-specific susceptibility patterns observed. The biological basis for such gender differences has been well documented in previous studies. Research has shown that androgens can significantly increase the expression of CYP4F2 and CYP4F3 through androgen receptor (AR) in prostate cancer cells (52), while PON1 activity is modulated by estrogen through HDL metabolism pathways (53). Furthermore, animal studies have demonstrated that male subjects show higher metabolic activation and sensitivity to organophosphate compounds (54), which may be attributed to the higher expression of male-specific CYP450 enzymes such as CYP2C11 and CYP3A1 (55). The significant positive correlation observed in younger populations (<60 years) likely reflects their higher metabolic enzyme activity and more efficient detoxification capacity, contrasting with age-related metabolic decline (56). This age-related difference in xenobiotic metabolism has been well documented, as aging is associated with reduced liver blood flow, decreased hepatic mass (57), and declined expression of specific CYP enzymes involved in xenobiotic metabolism (58). The enhanced effect in smokers relates to cigarette smoke polycyclic aromatic hydrocarbons activating AhR, increasing toxic metabolite production and exacerbating DNA damage and oxidative stress (59). Given the widespread environmental presence of OPPs and their significant biological effects, these findings have important public health implications for identifying high-risk populations and developing targeted prevention strategies.

However, several limitations should be acknowledged. The cross-sectional study design precludes the establishment of causal relationships between OPP exposure and AD. The reliance on single-point urinary OPP metabolite measurements represents another important limitation. Given that OPPs have relatively short biological half-lives ranging from hours to days (60), and their exposure patterns can vary substantially due to dietary choices and behavioral changes (61), single spot urine samples may not accurately reflect long-term exposure patterns. It is recognized that they may not fully capture the temporal variability in OPP exposure (62). Another important limitation is the reliance on self-reported questionnaires for AD diagnosis, which may introduce misclassification bias. Although the questionnaires used in NHANES underwent rigorous validation, the lack of clinical verification might lead to either over- or under-reporting of AD cases. Furthermore, we were unable to assess certain environmental risk factors such as occupational exposure to pesticides or other workplace-related chemical exposures, which might contribute to both OPP exposure levels and AD risk. While potential mechanisms involving inflammatory responses and immune modulation were proposed, the study did not directly investigate these biological pathways. Additionally, despite the comprehensive analysis, there might be unmeasured confounding factors that could influence the observed associations. Furthermore, no corrections for multiple comparisons were applied in our stratified analyses, which might increase the risk of Type I error. These limitations suggest the need for future longitudinal studies with repeated exposure measurements, as multiple urine samples collected over time would provide more reliable estimates of chronic exposure (63), along with mechanistic investigations to further validate these findings.

## Conclusion

In conclusion, this study demonstrates that OPP exposure, especially DMP, is associated with increased AD risk in adults, with stronger associations in males, younger individuals, and smokers. While mechanistic studies are needed, these findings highlight the role of environmental factors in AD development and suggest targeted prevention strategies for susceptible populations.

## Data Availability

Publicly available datasets were analyzed in this study. This data can be found at: data are accessible in a public, open access repository. Open access data can be found on the NHANES website: https://www.cdc.gov/nchs/nhanes/index.htm.
