# Supplementary material for: Association between organophosphate pesticide exposure and atopic dermatitis: a cross-sectional study based on NHANES 1999–2007
Source: Front Public Health. 2025 Mar 6;13:1555731. doi: 10.3389/fpubh.2025.1555731 (PMC11922850; doi:10.3389/fpubh.2025.1555731)
Supplement: Supplementary file 1 [file Table_1.DOCX]

Table 1. Baseline characteristics of AD population according to sex.

| Variables | Sex | | P-value |
| --- | --- | --- | --- |
|  | Male | Female |  |
| Age(years) | 44.02±1.44 | 40.55±1.10 | 0.050 |
| Poverty income ratio | 3.17±0.18 | 3.04±0.18 | 0.589 |
| ln(DMP, ug/L) | 0.76±0.17 | 0.39±0.12 | 0.085 |
| ln(DEP, ug/L) | 0.24±0.18 | -0.05±0.14 | 0.200 |
| ln(DMTP, ug/L) | 0.94±0.17 | 0.74±0.19 | 0.406 |
| ln(DETP, ug/L) | -0.48±0.12 | -0.62±0.13 | 0.382 |
| ln(DMDTP, ug/L) | -0.67±0.16 | -0.66±0.16 | 0.971 |
| ln(DEDTP, ug/L) | -1.44±0.10 | -1.44±0.11 | 0.955 |
| ln(Urinary creatinine, mg/dL) | 4.86±0.06 | 4.56±0.06 | 0.003 |
| DII | 0.60±0.16 | 1.78±0.17 | <0.001 |
| PA(hours/week) | 268.35±35.43 | 166.06±14.73 | 0.008 |
| Race |  |  | 0.704 |
| Mexican American | 13 (4.33%) | 17 (3.44%) |  |
| Other Hispanic | 3 (3.18%) | 9 (3.50%) |  |
| Non-Hispanic White | 63 (72.01%) | 101 (78.76%) |  |
| Non-Hispanic Black | 30 (12.84%) | 28 (8.22%) |  |
| Other Race | 8 (7.63%) | 8 (6.09%) |  |
| Education level |  |  | 0.046 |
| Less than 9th grade | 7 (4.44%) | 5 (1.36%) |  |
| 9-11th grade | 14 (8.89%) | 19 (8.35%) |  |
| High school graduate | 22 (20.26%) | 40 (27.71%) |  |
| College | 38 (29.58%) | 66 (40.13%) |  |
| College graduate or above | 36 (36.82%) | 33 (22.45%) |  |
| Smoke |  |  | 0.441 |
| No | 44 (42.94%) | 84 (47.63%) |  |
| Yes | 73 (57.06%) | 79 (52.37%) |  |
| Alcohol consumption |  |  | 0.007 |
| No | 14 (12.79%) | 48 (28.10%) |  |
| Yes | 98 (87.21%) | 101 (71.90%) |  |
| Hypertension |  |  | 0.084 |
| No | 81 (71.13%) | 129 (81.25%) |  |
| Yes | 36 (28.87%) | 34 (18.75%) |  |
| Diabetes |  |  | 0.714 |
| No | 111 (95.86%) | 157 (96.73%) |  |
| Yes | 6 (4.14%) | 6 (3.27%) |  |

**Abbreviations**: AD, atopic dermatitis; DMP, dimethylphosphate; DEP, diethylphosphate; DII, dietary inflammatory index; DMTP, dimethylthiophosphate; DETP, diethylthiophosphate; DMDTP, dimethyldithiophosphate; DEDTP, diethyldithiophosphate; PA, Physical activity.
